# Transcriptomic analysis of the seminal vesicle response to the reproductive toxicant acrylamide

**DOI:** 10.1186/s12864-021-07951-1

**Published:** 2021-10-08

**Authors:** David A. Skerrett-Byrne, Brett Nixon, Elizabeth G. Bromfield, James Breen, Natalie A. Trigg, Simone J. Stanger, Ilana R. Bernstein, Amanda L. Anderson, Tessa Lord, R. John Aitken, Shaun D. Roman, Sarah A. Robertson, John E. Schjenken

**Affiliations:** 1 Priority Research Centre for Reproductive Science, School of Environmental and Life Sciences, Discipline of Biological Sciences, The University of Newcastle, University Drive, Callaghan, NSW 2308 Australia; 2 Hunter Medical Research Institute, Pregnancy and Reproduction Program, New Lambton Heights, NSW 2305 Australia; 3 Department of Biochemistry and Cell Biology, Faculty of Veterinary Medicine, Utrecht University, 3584 CM Utrecht, The Netherlands; 4 The Robinson Research Institute and Adelaide Medical School, University of Adelaide, Adelaide, SA 5005 Australia; 5 South Australian Genomics Centre (SAGC), South Australian Health & Medical Research Institute (SAHMRI), Adelaide, SA 5000 Australia; 6 Computational & Systems Biology Program, Precision Medicine Theme, South Australian Health & Medical Research Institute (SAHMRI), Adelaide, SA 5000 Australia; 7 Adelaide Medical School, Faculty of Health & Medical Sciences, University of Adelaide, Adelaide, SA 5005 Australia

**Keywords:** Acrylamide, Reproduction, Reproductive toxicant, Seminal vesicle, Transcriptomics

## Abstract

**Background:**

The seminal vesicles synthesise bioactive factors that support gamete function, modulate the female reproductive tract to promote implantation, and influence developmental programming of offspring phenotype. Despite the significance of the seminal vesicles in reproduction, their biology remains poorly defined. Here, to advance understanding of seminal vesicle biology, we analyse the mouse seminal vesicle transcriptome under normal physiological conditions and in response to acute exposure to the reproductive toxicant acrylamide. Mice were administered acrylamide (25 mg/kg bw/day) or vehicle control daily for five consecutive days prior to collecting seminal vesicle tissue 72 h following the final injection.

**Results:**

A total of 15,304 genes were identified in the seminal vesicles with those encoding secreted proteins amongst the most abundant. In addition to reproductive hormone pathways, functional annotation of the seminal vesicle transcriptome identified cell proliferation, protein synthesis, and cellular death and survival pathways as prominent biological processes. Administration of acrylamide elicited 70 differentially regulated (fold-change ≥1.5 or ≤ 0.67) genes, several of which were orthogonally validated using quantitative PCR. Pathways that initiate gene and protein synthesis to promote cellular survival were prominent amongst the dysregulated pathways. Inflammation was also a key transcriptomic response to acrylamide, with the cytokine, *Colony stimulating factor 2* (*Csf2*) identified as a top-ranked upstream driver and inflammatory mediator associated with recovery of homeostasis. *Early growth response* (*Egr1*), *C-C motif chemokine ligand 8* (*Ccl8*), and *Collagen, type V, alpha 1* (*Col5a1*) were also identified amongst the dysregulated genes. Additionally, acrylamide treatment led to subtle changes in the expression of genes that encode proteins secreted by the seminal vesicle, including the complement regulator, *Complement factor b* (*Cfb*).

**Conclusions:**

These data add to emerging evidence demonstrating that the seminal vesicles, like other male reproductive tract tissues, are sensitive to environmental insults, and respond in a manner with potential to exert impact on fetal development and later offspring health.

**Supplementary Information:**

The online version contains supplementary material available at 10.1186/s12864-021-07951-1.

## Background

Paternal events and exposures prior to conception have biological effects extending beyond fertility to impact the development and health of subsequent generations [1–3]. While genetic and epigenetic modifications to sperm partly explain paternal programming effects, emerging evidence demonstrates that information is also transmitted to offspring via alterations in seminal plasma composition that affect sperm and interact with the female reproductive tract after mating [4–7].

Chief among the male accessory sex glands, secretions of the seminal vesicle are a major contributor to seminal plasma in most mammalian species [8, 9]. The primary function of the seminal vesicles is to synthesise and secrete a diverse array of bioactive factors, with functional roles in semen coagulation, regulation of sperm function, and modulation of the female reproductive tract immune response [5, 6, 9, 10]. Broadly speaking, these functions promote the likelihood of male reproductive success and are conserved across mammalian species, despite species differences in reproductive strategy and tissue anatomy [8–10].

Notably, seminal vesicle secretions exhibit high levels of plasticity in response to changing paternal environments [11–13]. Several studies report seminal vesicle responsiveness to paternal physiological disturbances and environmental insults such as metabolic disorder [14], nutritional deficiency [15], endocrine disrupting compound exposure [16], and heat or psychosocial stress [13, 17]. While seminal vesicle secretions are influenced by circulating androgens and estrogens, hormonal control appears to primarily regulate the secretory capacity of the seminal vesicles, as opposed to the composition of their secretions [18–21]. For example, male mice administered testosterone have increased seminal vesicle secretory activity, but their immune-stimulating activity in the female reproductive tract after mating remains unchanged [22], while social status in mice alters seminal vesicle secretory capacity and composition in a manner thought to be independent of androgens [23]. These data imply that other as yet uncharacterised factors exert influence on the composition of seminal vesicle secretions [6, 24] and thus highlight a pressing need for additional research to characterise seminal vesicle physiology.

We have recently begun to address this knowledge gap by applying advanced proteomic and bioinformatics platforms to provide greater insight into the complexity of mouse seminal vesicle function [2]. In these studies, we utilised a well-established acute acrylamide exposure model [25–29] to demonstrate that the seminal vesicles rapidly respond to this model reproductive toxicant in a manner consistent with imparting consequences for fetal development and later offspring health. The exposure regimen, which featured administration of supra-physiological concentrations of acrylamide, elicited substantially increased rates of fetal loss without an attendant reduction in sperm fertilising ability [25]. Our findings also provide evidence that the male transfers the burden of acrylamide stress to the female reproductive tract, through mechanisms that are independent of sperm DNA damage [25]. Since the nature of these paternal stress signals and the timing of their transfer to spermatozoa remain unresolved, here we performed RNA-seq analysis to assess the seminal vesicle tissue transcriptome under normal physiological conditions, and to determine the extent of dysregulation resulting from acute acrylamide exposure. This strategy offers the promise of enhancing understanding of mouse seminal vesicle tissue physiology and identifying key molecular pathways that regulate its response to paternal stressors [30].

## Results

### Global transcriptomic analysis of mouse seminal vesicles

**Fig. 1:**
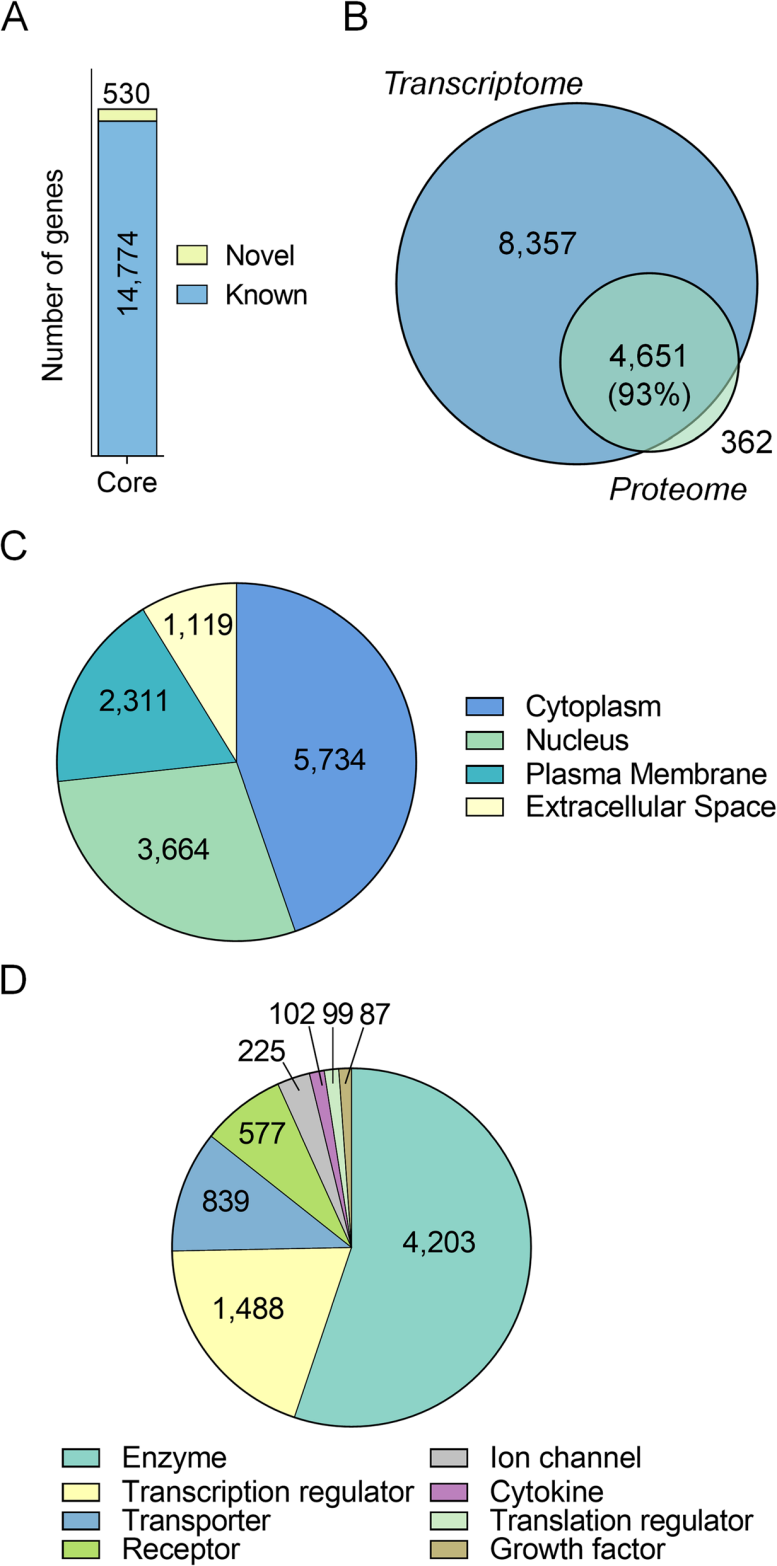
Characterisation and functional annotation of the mouse seminal vesicle transcriptome. **A** Genes detected in the seminal vesicle were determined using the average normalised expression values calculated using DeSeq2. Expression levels were filtered to remove lowly abundant genes with an average expression ≤1. Detected genes were further separated into novel (yellow) or known (blue). Numbers presented within the column represent the number of genes within each category. **B** Genes detected in the mouse seminal vesicle were further compared to seminal vesicle proteomic data using a Venn diagram. Functional annotation of the seminal vesicle transcriptome using Ingenuity Pathway Analysis revealed the predicted **C** cellular localisation and **D** biological function of the detected genes. All graphical components were generated using Prism (version 9.0.0)

Initially, we undertook a transcriptomic characterisation of mouse seminal vesicles using the DNBSeq platform. Transcriptomic analysis of control seminal vesicle tissue returned a complex core transcriptome of 17,258 genes (Additional file 1, Table A1). Filtering to remove lowly abundant genes (< 1 average normalised expression across sample replicates (DESeq2)) resulted in a final set of 15,304 genes for downstream analyses. Each replicate had high correlation coefficients (0.976 average, Additional file 2, Fig. A1)*.* The majority of the transcriptome consisted of known genes (14,774, 97%), but additionally uncovered 530 (3%) previously unidentified, predicted novel genes outside the regular mouse gene annotation using Cufflinks and Coding Potential Calculator (CPC) (Fig. 1A; Additional file 1, Table A1). Additionally, 13,008 of the 15,304 identified genes mapped to reviewed proteins from UniProt, and of these, 4651 were detected in our recent mouse seminal vesicle proteomic analysis (93% coverage of proteomic data) (Fig. 1B). Notably, using data from the Mouse Genome Informatics Phenotypes/Alleles project and the International Mouse Phenotyping Consortium, 431 of the genes detected in our seminal vesicle transcriptomic analysis have previously been associated with either male fertility, or seminal vesicle phenotypes (Additional file 1, Table A1).

To gain an overview of the cellular localisation and potential functions of the genes expressed in mouse seminal vesicles, we utilised Ingenuity Pathway Analysis (IPA) [15], which successfully annotated 14,773 of the 15,304 (97%) genes identified in control seminal vesicle tissue. The dominant cellular localisation assigned to the encoded gene products was cytoplasm (5734 genes, 39%), followed by nucleus (3664 genes, 25%), plasma membrane (2311 genes, 16%) and extracellular space (1119 genes, 8%) (Fig. 1C), with 1945 genes (12%) not assigned a specific category (data not shown). Classification of the functional category to which each gene product was assigned revealed that the most common categories were enzymes (4203, 28%), followed by transcription regulators (1488 genes, 10%), transporters (839 genes, 6%), receptors (577 genes, 4%) and ion channels (225 genes, 2%) (Fig. 1D). Genes classified as cytokines, translation regulators, and growth factors constituted less than 1% of the transcriptome, while 7153 (48%) were not assigned a functional category and are not presented in this analysis.

### Genes that encode secreted proteins are amongst the most abundant genes in the seminal vesicle

**Fig. 2:**
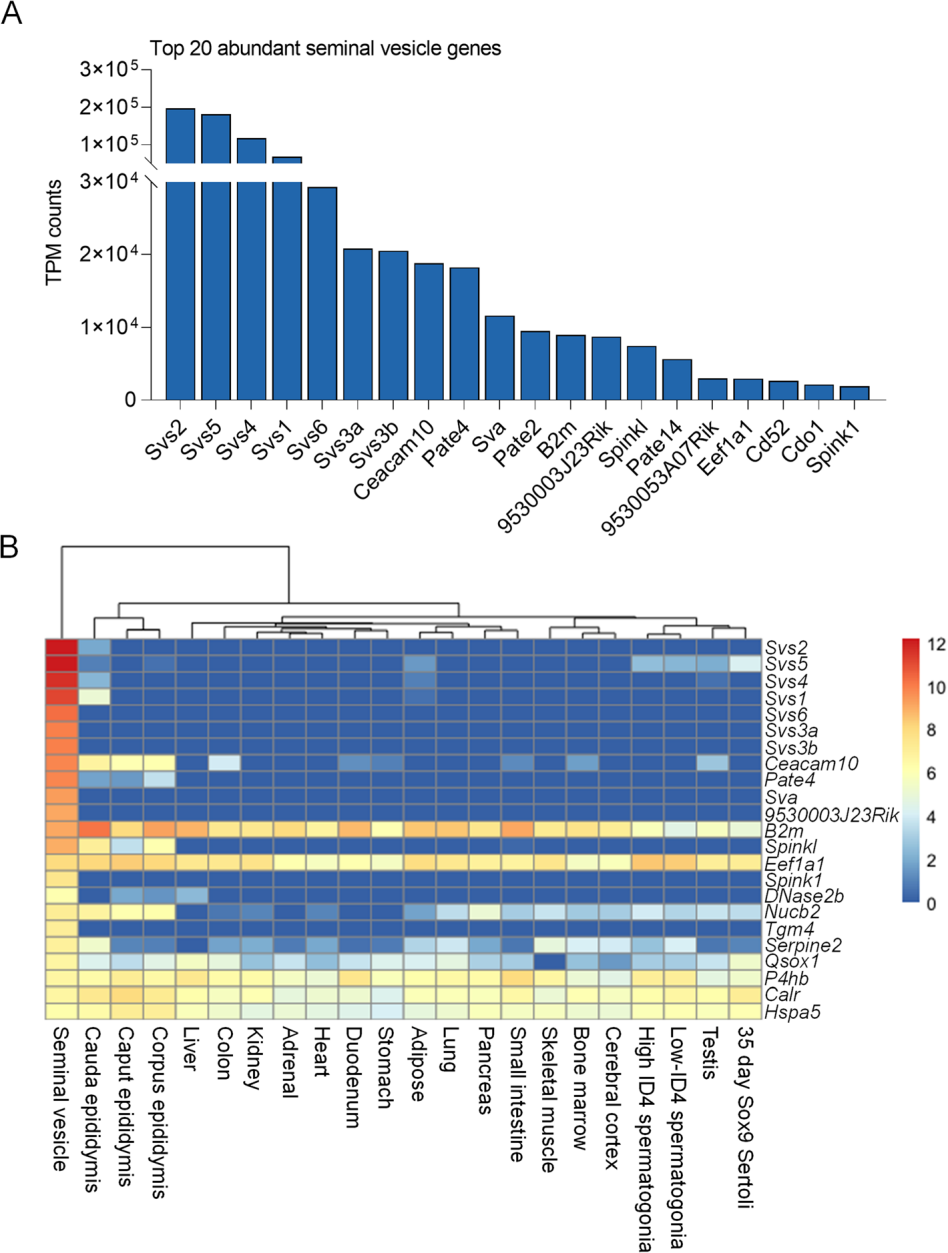
Genes that encode secreted proteins are amongst the most abundant genes in the seminal vesicle **A** Seminal vesicle genes were filtered based on their transcript per million (TPM) expression level and the 20 most abundant seminal vesicle genes are ranked and presented as a column graph. **B** Highly abundant seminal vesicle secreted proteins were then compared to existing transcriptomics datasets to determine expression levels across a variety of tissue types. Data are presented as a heat map using log2 calculated TPM expression levels and show expression of: Seminal vesicle, Epididymis (caput, corpus, cauda), Liver, Colon, Kidney, Adrenal gland, Heart, Duodenum, Stomach, Adipose tissue, Lung, Pancreas, Small intestine, Skeletal muscle, Bone marrow, Cerebral cortex, high ID4 Spermatogonia, low ID4 Spermatogonia, Testis, and Sertoli cells

Taking into account that the primary function of seminal vesicle tissue is to produce and secrete bioactive factors that influence both male gametes and female reproductive tract function [6, 9], we next compared the genes identified in seminal vesicle tissue with that of proteins detected in seminal vesicle fluid. To achieve this, we initially curated the seminal vesicle transcriptome based upon the average gene expression (transcripts per million, TPM) and collated proteomic data of seminal vesicle fluid from three published proteomic datasets [23, 31, 32]. Seminal vesicle fluid proteins were mapped to their corresponding mouse annotation resulting in a final list of 81 genes. Of these, 70/81 (86%) were detected in the seminal vesicle transcriptome (Additional file, Table A2). Amongst the top 20 most abundant genes, 15 (75%, Fig. 2A; Additional file, Table A2), encode proteins secreted by the seminal vesicles with seven identified as members of the *seminal vesicle secretory protein (Svs)* family, which play a crucial role in copulatory plug formation [24]. Additionally, 23/81 (28%) ranked in the top 50 of the most abundant seminal vesicle as measured based upon average TPM genes (Fig. 2B).

To compare the specificity of the seminal vesicle transcriptome with that of other mouse tissues, we interrogated data from a recent large scale discovery study of male reproductive tract specific genes [33] (Fig. 2B). Using this approach in combination with hierarchical clustering, highly abundant gene products secreted from the seminal vesicles formed a distinct cluster with uniquely high expression of *Svs2*, *Svs5*, *Svs4*, *Svs1*, *Svs6*, *Svs3a*, *Svs3b*, *Seminal vesicle antigen* (*Sva*), *RIKEN cDNA 9530003J23 gene* (*9530003J23Rik*), *Serine peptidase inhibitor, Kazal type 1* (*Spink1*), *Deoxyribonuclease II beta* (*Dnase2b*) and *Transglutaminase 4* (*Tgm4*). Notably, while the seminal vesicles exhibited the highest expression of *Carcinoembryonic antigen-related cell adhesion molecule 10* (*Ceacam10*), *Prostate and testis expressed 4* (*Pate4*), *and Serine protease inhibitor, Kazal type-like* (*Spinkl*), high expression was also observed in other reproductive tract tissues including the epididymis (*Ceacam10, Pate4, Spinkl*). By contrast, genes such as *Beta 2 microglobulin* (*B2m*)*, Eukaryotic translation elongation factor 1 alpha 1* (*Eef1a1*)*, Nucleobindin 2* (*Nucb2*)*, Serine (or cysteine) peptidase inhibitor, clade E, member 2* (*Serpine2*)*, Quiescin Q6 sulfhydryl oxidase 1* (*Qsox1*)*, Prolyl 4-hydroxylase, beta polypeptide* (*P4hb*)*, Calreticulin* (*Calr*)*,* and *Heat shock protein 5* (*Hspa5)* showed broad expression across tissue types (Fig. 2B).

### Functional characterisation of mouse seminal vesicle genes using ingenuity pathway analysis

**Fig. 3:**
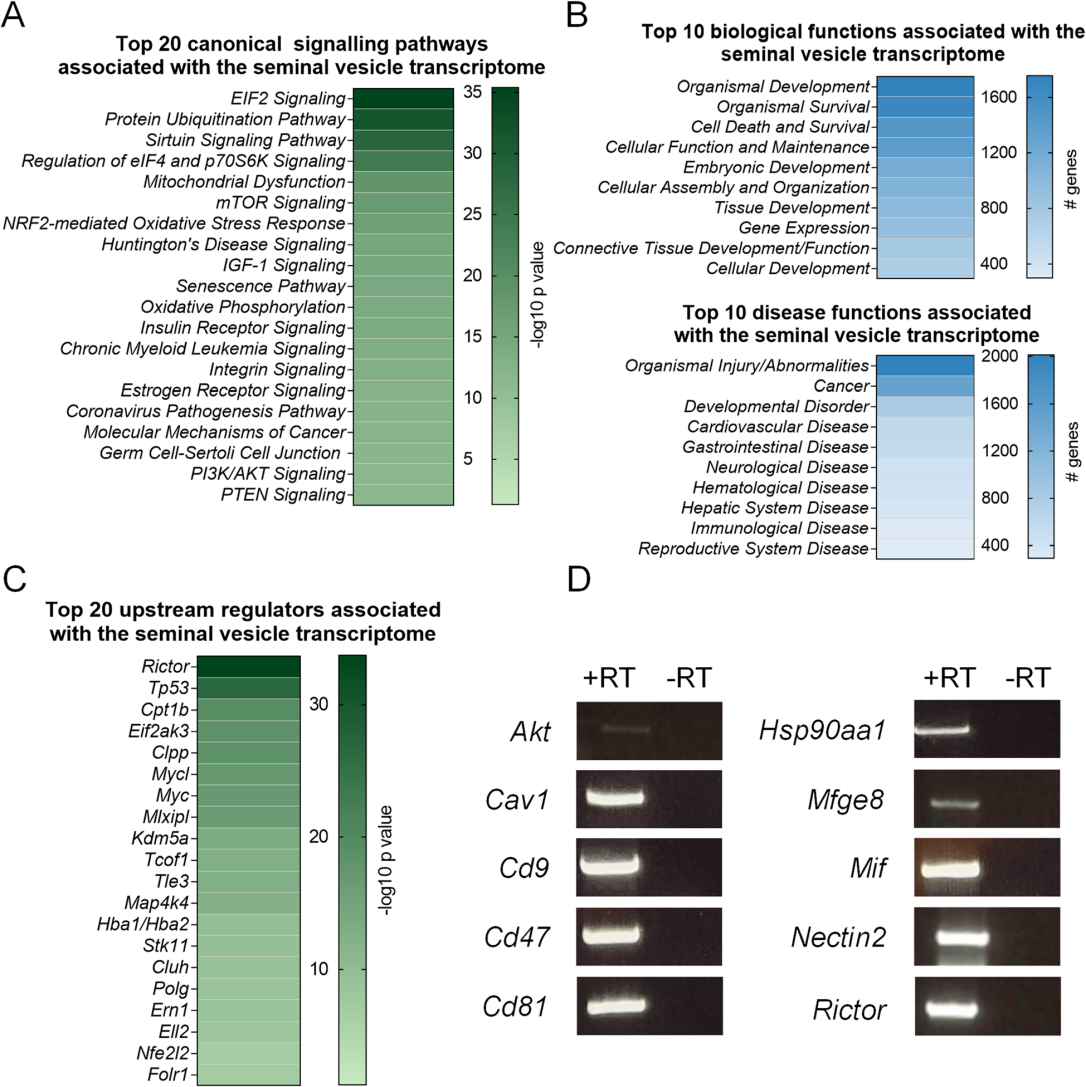
Functions of genes expressed in the mouse seminal vesicle. **A-C** Ingenuity Pathway Analysis was used to annotate the core seminal vesicle transcriptome based on canonical signalling pathways, biological and disease functions, and upstream regulators of the expressed genes that were highly (*p* ≤ 0.05) predicted to be involved in mouse seminal vesicle function. Heat maps depict the top **A** 20 canonical signalling pathways based on –log10 *p* values, **B** 10 biological and disease functions based on number of target molecules in dataset, and **C** 20 upstream regulators based on –log10 *p* values. **D** A subset of ten genes from the mouse seminal vesicle transcriptome were selected for conventional PCR confirmation (*n* = 4). Representative agarose gel electrophoresis images of *Thymoma viral proto-oncogene 1* (*Akt*), *Caveolin 1* (*Cav1*), *Cluster of differentiation* (*Cd*)*9 antigen* (*Cd9*), *CD47 antigen* (*Cd47*), *CD81 antigen* (*Cd81*), *Heat shock protein 90, alpha (cytosolic), class A member 1* (*Hsp90aa1*), *Nectin cell adhesion molecule 2* (*Nectin2*), *Milk fat globule-EGF factor 8 protein* (*Mfge8*), *Macrophage migration inhibitory factor* (*Mif*) and *Rapamycin-insensitive companion of mTOR* (*Rictor*) detection with equivalent reverse transcription negative controls*.* Gel images are presented as cropped images for clarity and conciseness with corresponding full length gel images provided in Additional file 2, Figure A2

IPA was next used to predict canonical pathways, upstream regulators and downstream molecular and cellular, and physiological system functions associated with genes expressed in the mouse seminal vesicle (Fig. 3A-C; Additional file 1, Table A3–5). As IPA is limited to the curation of a maximum of 8000 molecules in any one analysis, we restricted our assessment to those core genes with a normalised expression across sample replicates (DESeq2) > 150 (comprising 7554 analysis ready genes). Given that reproductive hormones are pivotal in all aspects of seminal vesicle development and function [10, 18, 19], we initially performed a targeted analysis and explored the association of seminal vesicle genes with endocrine function. Indeed, within the seminal vesicle transcriptome, evidence of the hormonal responsiveness of this tissue was observed with estrogen receptor signalling (189 genes, 58.9% coverage), and androgen signalling (71 genes, 53.8% coverage) pathways identified (Fig. 3A; Additional file 1, Table A3). Further, genes associated with endocrine system disorders (91 genes) and other reproductive functions, including embryonic development (1174 genes), reproductive system development and function (124 genes), and reproductive system disease (293 genes) (Fig. 3B; Additional file 1, Table A4) were among the functions prominently associated with the mouse seminal vesicle transcriptome.

To explore alternative seminal vesicle gene functions, we next conducted an unbiased analysis of the seminal vesicle transcriptome. This strategy identified canonical pathways associated with cellular proliferation, protein synthesis and cellular stress (Fig. 3A; Additional file 1, Table A3) as being prominently represented. Specific examples of these pathways included eukaryotic initiation factor (EIF)2 (167 genes, 81.5% coverage), protein ubiquitination (196 genes, 74.8% coverage), mitochondrial dysfunction (118 genes, 73.8% coverage), mammalian target of rapamycin (mTOR) (137 genes, 69.2% coverage), and nuclear factor erythroid 2-related factor-2 (NRF2)-mediated oxidative stress response (125 genes, 69.8% coverage). Other highly predicted functions associated with seminal vesicle genes were cell proliferation/protein production (gene expression: 909 genes; cellular growth and proliferation: 666 genes; and protein synthesis: 355 genes), and cellular stress (organismal survival: 1661 genes; cell death and survival: 1459 genes; immunological disease; 299 genes, cellular compromise; 174 genes; free radical scavenging; 89 genes inflammatory response; 40 genes, infectious disease; 36 genes) (Fig. 3B; Additional file 1, Table A4).

To predict canonical regulators that may dictate normal seminal vesicle function, we also used the upstream regulator function of IPA. Consistent with our earlier data (Fig. 3A-B) prominent upstream regulators were associated with cell proliferation and protein synthesis. These included, *Rapamycin-insensitive companion of mTOR* (*Rictor*, *p* = 1.96E-34, 201 targets), *Transcriptional regulator tumor protein p53* (*Tp53*, *p* = 1.68E-27, 601 targets), *Myc proto-oncogene* (*Myc*, *p* = 1.54E-17, 164 targets) and *Mlx interacting protein like* (*Mlxipl*, *p* = 4.65E-17, 103 targets) (Fig. 3C; Additional file 1, Table A5). When significant upstream regulators were ranked by the number of associated seminal vesicle genes, regulators with immune function were highly predicted, with *Interleukin* (*Il*)*4* (*p* = 1.39E-05, 197 targets), *Adp-ribosyl cyclase/cyclic adp-ribose hydrolase 1* (*Cd38*, *p* = 2.87E-05, 83 targets), and *Il5* (*p* = 1.80E-05, 77 targets) all identified (Additional file 1, Table A5).

Based on these analyses, a subset of ten genes with IPA mapped functions relating to protein synthesis, cellular stress, reproduction, and immune function (Additional file 1, Table A3–5) were selected for confirmation of transcriptomic data (Fig. 3D; Additional file 2, Fig. A2). Each of these ten targets (*Thymoma viral proto-oncogene 1* (*Akt*), *Caveolin 1* (*Cav1*), *Cluster of differentiation (Cd)9 antigen* (*Cd9*), *CD47 antigen* (*Cd47*), *CD81 antigen* (*Cd81*), *Heat shock protein 90, alpha (cytosolic), class A member 1* (*Hsp90aa1*), *Milk fat globule-EGF factor 8 protein* (*Mfge8*), *Macrophage migration inhibitory factor* (*Mif*), *Nectin cell adhesion molecule 2* (*Nectin2*), and *Rictor*), were confirmed by conventional PCR to be expressed in mouse seminal vesicle tissue.

### Acute acrylamide exposure alters the seminal vesicle transcriptome

To assess the impact of acrylamide exposure on the seminal vesicle transcriptome, male mice were administered daily injections of acrylamide (25 mg/kg bw/day) for five consecutive days using an established acute exposure model [25–27]. Global analysis of the mouse seminal vesicle transcriptome from acrylamide treated males revealed strong correlation coefficients (average of 0.973), indicative of a consistent response to this insult across all biological replicates (Additional file 2, Figure A1).

**Fig. 4:**
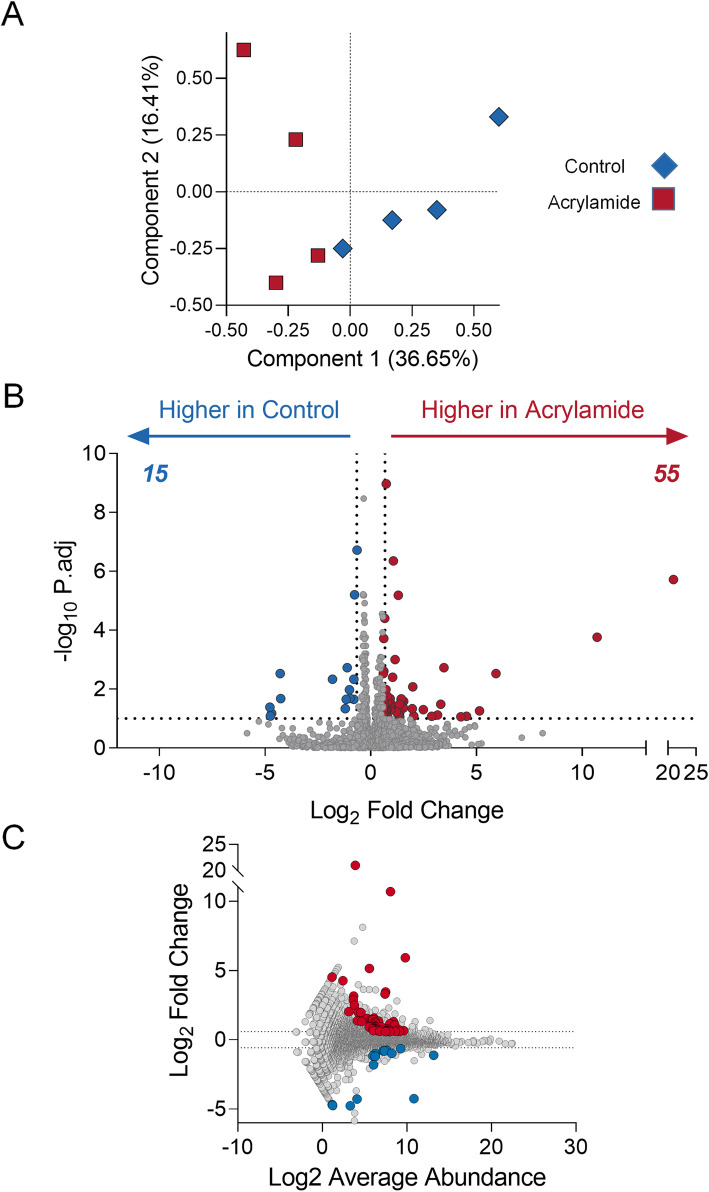
Acrylamide exposure alters the mouse seminal vesicle transcriptome. **A** Differentially regulated genes in the mouse seminal vesicles were identified following acrylamide treatment using DEseq2 and were filtered based on those genes with a fold-change ≥1.5 or ≤ 0.67 and an adjusted p (*p.adj*) value ≤0.1. These data are represented using **A** Principal Component Analysis plot of transcriptomic data from acrylamide (red) and control (blue) samples to identify transcriptome wide changes across biological replicates, and **B-C** plots to identify specific genes that meet the fold-change and *p.adj* threshold criteria. **B** Volcano plot comparing fold-change and –Log10 p.adj, and **C** MA plot comparing Log2 average abundance to Log2 fold-change. Red data points indicate genes that are higher in acrylamide treated samples, blue data points indicates genes higher in control samples, grey data points indicate genes that do not meet the fold-change and *p.adj* criteria. All graphical components were generated using Prism (version 9.0.0)

Initially, unsupervised clustering of gene expression profiles using Principal Component Analysis (PCA) demonstrated groupings in the global gene expression profile consistent with treatment group, separating on the first PC that contains the larger proportion of variability (36.65%) (Fig. 4A). Combined analysis of control and acrylamide transcriptomes using DESeq2 to identify differentially regulated genes, demonstrated that a subset of genes was altered in response to acrylamide exposure, with 55 up-regulated and 15 down-regulated (fold-change ≥1.5 or ≤ 0.67 and a false discovery rate adjusted *p* value (*p.adj*) ≤ 0.1) (Fig. 4B; Additional file 1, Table A1). These differentially regulated genes varied in abundance, although none ranked among the most highly expressed seminal vesicle genes (Fig. 4C; Additional file 1, Table A1).

Among the ten most differentially up- and down-regulated genes (Table 1), common functions ascribed were: immune response [*C-C motif chemokine (Ccl)21a, Indoleamine 2,3-dioxygenase 1* (*Ido1*)*, Cathepsin E* (*Ctse*)*, Early growth response* (*Egr*)1*, Egr2, Deleted in malignant brain tumors 1* (*Dmbt1*)*, Defensin alpha 24* (*Defa24*)*, Intelectin 1* (*Itln1*)*,* and *PILR alpha associated neural protein* (*Pianp*)] [34–41], cell proliferation and transcription/protein synthesis [*Rpl29, Egr1, Egr2, Sterile alpha motif domain containing 11* (*Samd11*)*, D-2-Hydroxyglutarate dehydrogenase* (*D2hgdh*)*,* and *Ribosomal protein SA* (*Rpsa*)] [41–44], cellular stress and survival [*Egr1,* and *Poly (ADP-Ribose) Polymerase 1 binding protein* (*Parpbp*)] [45, 46], and sperm function [*Dynein axonemal heavy chain 1* (*Dnah1*)] [47]. In addition, five of the top up and down differentially regulated genes were novel or Riken genes with yet to be characterised functions (Table 1). Notably, 6/70 (9%) of the differentially regulated genes were associated with reproductive phenotypes identified from the Mouse Genome Informatics Phenotypes/Alleles project and the International Mouse Phenotyping Consortium projects (Additional file 1, Table A1). These genes included *Egr1* (9.84-fold increase, *p.adj =* 3.23E-02), and *B cell leukemia/lymphoma 6* (*Bcl6,* 0.58-fold decrease, *p.adj =* 6.52E-06) and were associated with mouse fertility phenotypes.

**Table 1:** List of the top 10 up- and down-regulated seminal vesicle genes following acute acrylamide exposure

**Up regulated**		
**Symbol**	**Fold-change (Acrylamide/Control)**	***p.adj***
*Ccl21a*	2,095,824.43	1.92E-06
*BGI_novel_G000684*	1660.02	1.73E-04
*Rpl29*	60.61	2.92E-03
*Ido1*	35.15	5.43E-02
*Dnah1*	22.91	8.30E-02
*Parpbp*	19.22	8.66E-02
*Ctse*	10.93	1.88E-03
*Egr1*	9.84	3.23E-02
*Egr2*	8.87	7.61E-02
*Samd11*	7.30	8.31E-02
**Downregulated**		
**Symbol**	**Fold-change (Acrylamide/Control)**	***p.adj***
*BGI_novel_G000643*	0.04	4.05E-02
*Dmbt1*	0.04	8.14E-02
*Defa24*	0.04	6.68E-02
*Itln1*	0.05	2.92E-03
*BGI_novel_G000675*	0.05	2.10E-02
*1500015O10Rik*	0.28	4.59E-03
*D2hgdh*	0.43	4.58E-02
*Pianp*	0.45	2.23E-02
*Rpsa*	0.46	1.88E-03
*BGI_novel_G000111*	0.50	1.04E-02

**Fig. 5:**
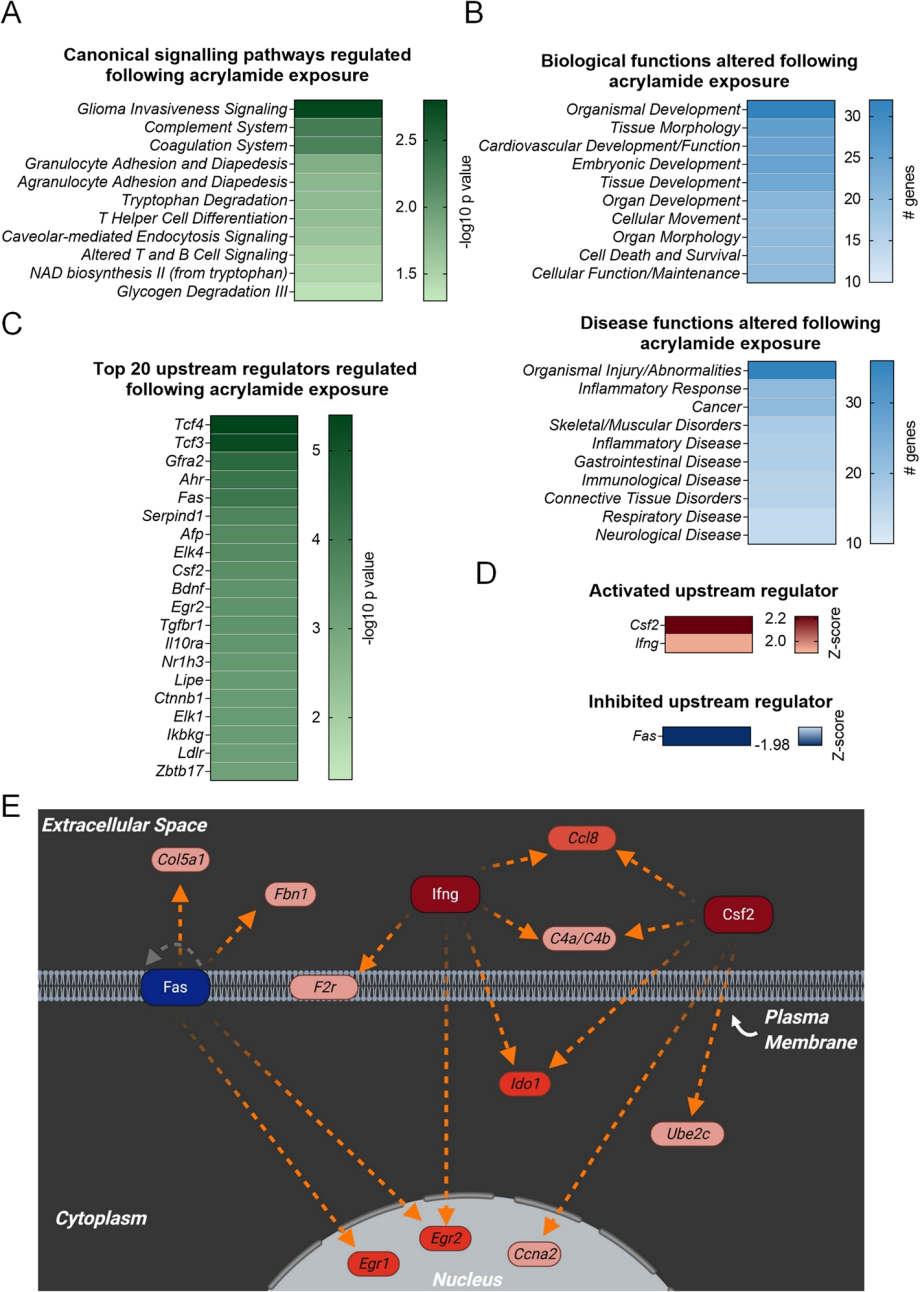
Functional characterisation of genes differentially regulated following acrylamide exposure. Ingenuity Pathway Analysis was used to annotate genes that were differentially regulated (fold-change ≥1.5 or ≤ 0.67 and an adjusted p (*p.adj*) value ≤0.1) following acrylamide treatment. Heat maps depict the top **A** canonical signalling pathways based on –log10 *p* values, **B** 10 biological and disease functions based on number of target molecules in dataset, and **C** 20 upstream regulators based on –log10 *p* values. To explore their regulation, **D** upstream regulators that were predicted to be activated (Z-score ≥ 2) or inhibited (Z-score ≤ − 2) with *p ≤* 0.05 are represented as heat maps based on Z-score value. **E** Differentially expressed genes following acrylamide exposure (red = up regulated) that are documented to be regulated by key activated (red central molecule) or inhibited (blue central molecule) upstream regulators are presented in an interaction network taking into account cellular localisation. Connecting lines indicate predicted relationships that lead to activation (orange), and relationship is known but effects on function is yet to be completely characterised (gray). Initial construction of the network was created in Ingenuity Pathway Analysis and schematics were redrawn using BioRender software (BioRender). Heatmaps were generated using Prism (version 9.0.0)

Immune response signalling pathways were prominent among the differentially expressed genes assessed by IPA. Notably, 5/11 of the predicted canonical pathways were associated with regulation of the immune response, although due to the small number of genes that were altered, Z-score predictions could not be calculated (Fig. 5A; Additional file 1, Table A6). Consistent with inflammation being a major function of the transcriptional response to acrylamide, molecular, cellular, and physiological system development functions, including immune cell trafficking (14 genes), cell-mediated immune response (7 genes) and humoral immune response (7 genes), were among the top molecular and physiological functions associated with the subset of differentially regulated genes (Fig. 5B; Additional file 1, Table A7). Similarly, pathways associated with inflammatory response (22 genes), inflammatory disease (17 genes), immunological disease (16 genes), and infectious disease (4 genes), were all altered in seminal vesicles following acrylamide treatment (Fig. 5B; Additional file 1, Table A7).

As observed in our global bioinformatic analysis of the mouse seminal vesicle transcriptome, acrylamide treatment also caused gene expression changes consistent with altered synthesis and production of proteins following cellular damage, including cell death and survival (20 genes), cellular growth and proliferation (16 genes), protein synthesis (4 genes), and gene expression (4 genes) (Fig. 5B; Additional file 1, Table A7). Additional relevant biological functions associated with the differentially regulated genes included embryonic development (25 genes), reproductive system development and function (4 genes), and reproductive system disease (4 genes) (Fig. 5B; Additional file 1, Table A7).

Application of the IPA upstream regulator prediction tool identified 218 molecules putatively involved in regulating the response of the seminal vesicles to acute acrylamide exposure (Fig. 5C; Additional file 1, Table A8), with one of these regulators, *Colony stimulating factor-2* (*Csf2*, Z-score = 2.22, *p =* 2.9E-04), reaching the Z-score threshold associated with activation. An additional two regulators were observed to be trending towards the Z-score threshold, *Interferon gamma* (*Ifng*, Z-score = 1.93, *p* = 2.88E-02) and conversely *Fas cell surface death receptor* (*Fas*, Z-score = − 1.98, *p* = 7.45E-05), suggestive of activation and inhibition respectively. However, in both cases, the Z-scores were marginally below the required threshold of ±2 to be considered robust candidates for regulating acrylamide responsive pathways in the seminal vesicles (Fig. 5C-E; Additional file 1, Table A8).

**Fig. 6:**
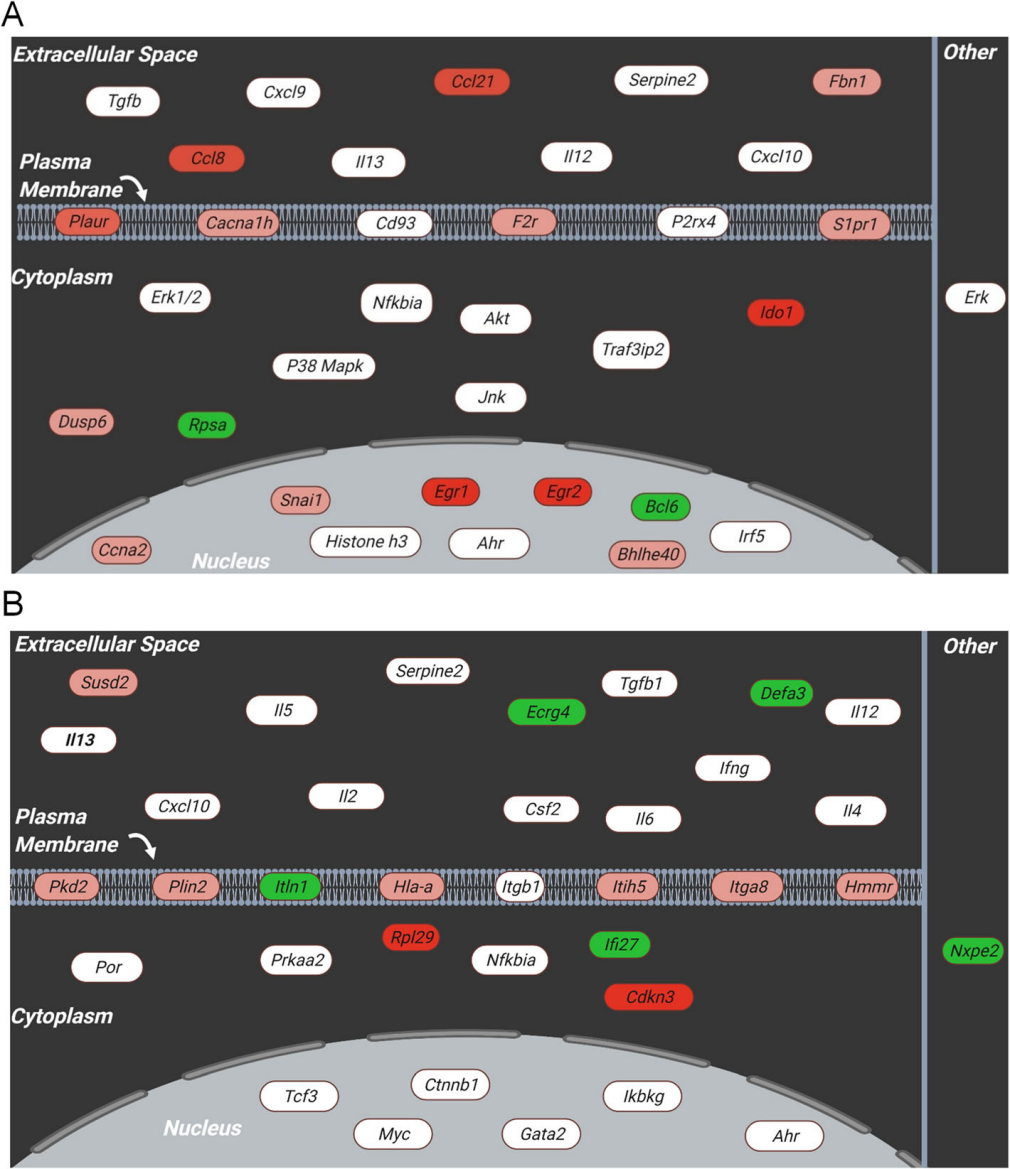
Acrylamide exposure leads to the activation of immune-associated gene networks. **A**, **B** Ingenuity Pathway Analysis was used to construct gene interaction networks taking into account cellular localisation that connected key genes (white) with differentially regulated genes (red = up-regulated, green = down-regulated) and enriched categories of diseases and functions. Networks predicted from these data include **A** haematological system development (16 differentially regulated genes), and **B** haematological system development and function and immune cell trafficking (14 differentially regulated genes). Initial construction of the network was created in Ingenuity Pathway Analysis and schematics were redrawn using BioRender software (BioRender)

Additional causal network analysis of the differentially regulated genes highlighted a number of gene networks in the seminal vesicles that were influenced by acrylamide exposure (Fig. 6). These networks were associated with two primary functions, namely: haematological system development and function (Fig. 6A*,* 16 focus molecules), and hematological system development and function and immune cell trafficking (Fig. 6B, 14 focus molecules). These functions reinforce the notion that a key transcriptomic response to acrylamide exposure is modulation of seminal vesicle genes linked to activation and regulation of the immune response.

**Fig. 7:**
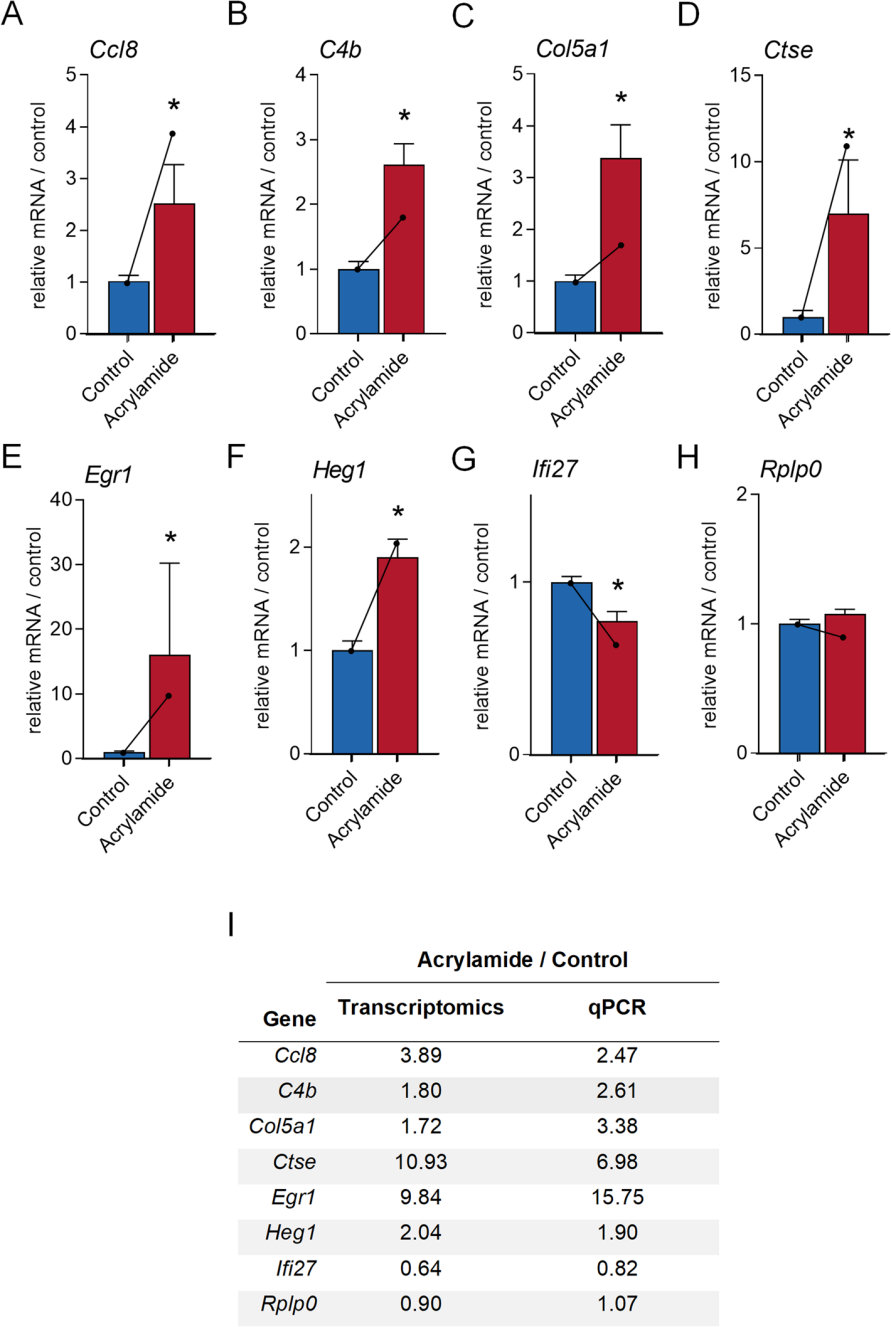
Orthogonal validation of genes differentially regulated following acrylamide exposure in the mouse seminal vesicles. RNA-sequencing data was validated using quantitative PCR on a selection of 8 genes identified in the mouse seminal vesicle transcriptome (*n* = 4–6 individual mice per treatment group). Candidate genes were selected based on those differentially regulated and associated with key canonical pathways and upstream regulators, including **A** *C-C motif chemokine (Ccl8),* **B** *Complement component 4b (C4b),* **C** *Collagen, type V, alpha 1 (Col5a1),* **D** *Cathepsin E (Ctse),* **E** *Early growth response 1 (Egr1),* **F** *Heart development protein with EGF-like domains 1 (Heg1)* **G** *Interferon alpha-inducible protein 27 (Ifi27),* and **H** *Ribosomal protein, large, P0 (Rplp0).* Quantitative PCR data was processed using the delta C(t) method using *Peptidyl-prolyl cis-trans isomerase A (Ppia)* as the reference gene and then normalised to the control group. Data are presented as a column graphs with mean ± SEM values with corresponding transcriptomics data overlaid onto these data using a line graph. qPCR data were analysed by non-parametric Mann Whitney U test. * *p* ≤ 0.05 indicates statistical significance when comparing acrylamide to control. **I** Table of qPCR validated genes showing direct comparison between RNA-sequencing fold-changes and qPCR fold-changes. All graphical components were generated using Prism (version 9.0.0)

To confirm our RNA-sequencing data, we validated the differential expression of a subset of genes that were dysregulated by acrylamide treatment using quantitative PCR (*n* = 4–6 per group). These included dysregulated genes with known functions of; tissue repair and remodelling; *Collagen, type V, alpha 1* (*Col5a1*, 1.72-fold increase, *p.adj* = 1.79E-02), and *Heart development protein with EGF-like domains 1* (*Heg1,* 2.04-fold increase, *p.adj* = 3.97E-03); immune response functions; *Cathepsin E (Ctse,* 10.93-fold increase, *p.adj* = 1.88E-03*)*, *C-C motif chemokine ligand 8* (*Ccl8,* 3.89-fold increase, *p.adj* = 4.57E-02), *Complement component 4b* (*C4b,* 1.80-fold increase, *p.adj* = 2.62E-02), *Egr1* (9.84-fold increase, *p.adj* = 3.23E-02) and *Interferon alpha-inducible protein 27* (*Ifi27*, 60% decrease, *p.adj* = 1.90E-07); and to demonstrate unaltered genes *Ribosomal protein, large, P0* (*Rplp0*, *0.90*-fold increase, *p.adj* = 2.57E-01) (Additional file 1, Table A1). Consistent with the transcriptomic data, Ccl8 (Fig. 7A, 2.47-fold increase, *p* = 4.0E-03), C4b (Fig. 7B, 2.61-fold increase, *p* = 4.3E-03), Col5a1 (Fig. 7C¸ 3.38-fold increase, *p* = 7.9E-03), Ctse (Fig. 7D¸ 6.98-fold increase, *p* = 9.0E-03), Egr1 (Fig. 7E, 15.75-fold increase, p = 9.0E-03), Heg1 (Fig. 7F, 1.90-fold increase, *p* = 8.7E-03) were induced, while Ifi27 (Fig. 7G, 24% decrease, *p* = 1.59E-02) was suppressed following acrylamide exposure (Fig. 7A-G). Genes that were not altered in the transcriptomic dataset, including *Ribosomal protein, large, P0* (*Rplp0, p.adj* = 2.75E-01) were similarly not altered following acrylamide exposure in the qPCR confirmation data (Fig. 7H; Additional file 1, Table A1).

### Acute acrylamide exposure alters the expression of seminal vesicle genes that encode secreted proteins

**Fig. 8:**
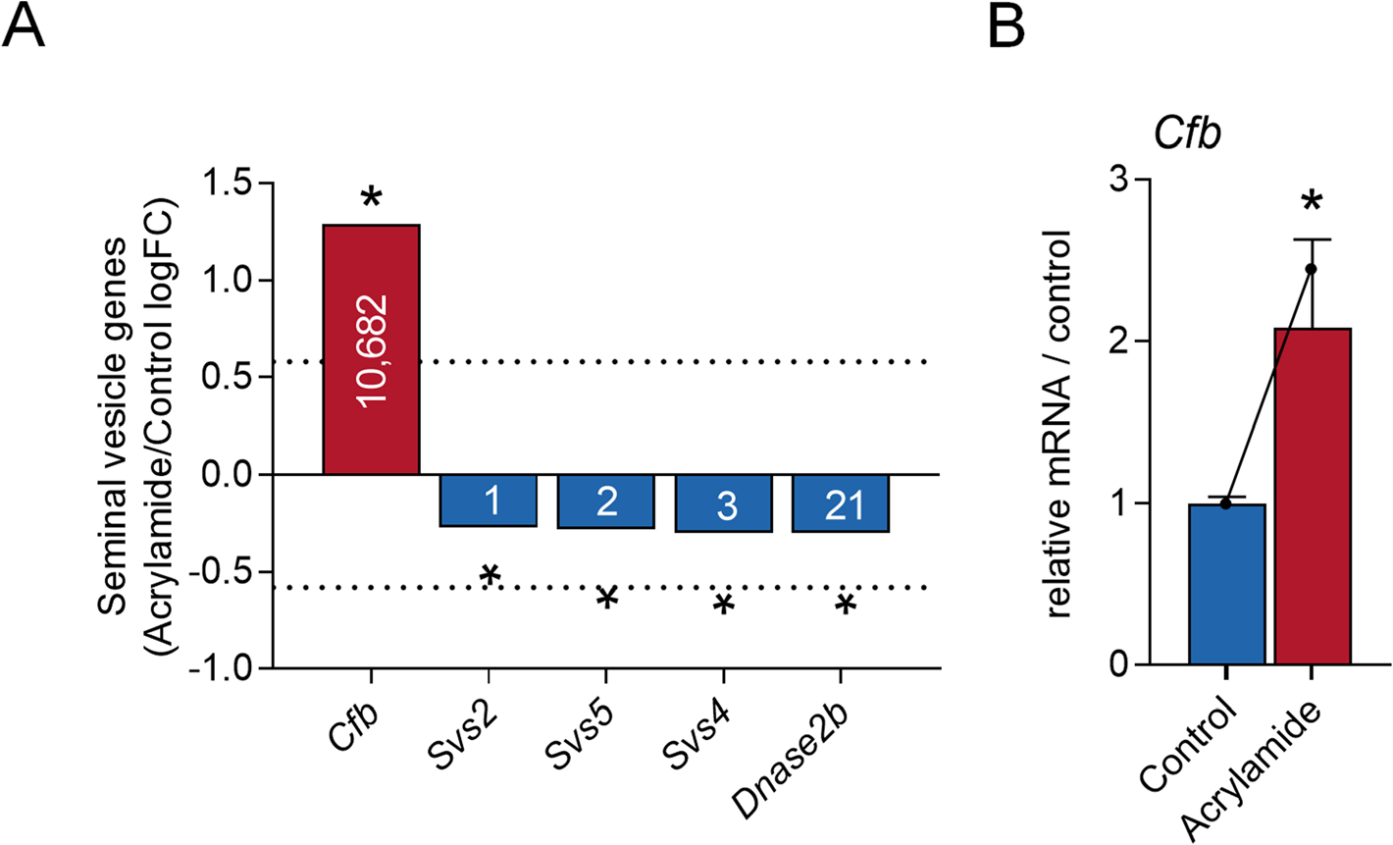
Genes that encode seminal vesicle secreted proteins are dysregulated following acrylamide exposure. **A** Expression of seminal vesicle secreted genes (*n* = 4 individual mice per treatment group) that were identified as statistically significant (adjusted p (*p.adj)* ≤ 0.1 indicated by *) in our transcriptomic analysis. Genes assessed included: *Complement factor b (Cfb)*, *Seminal vesicle secretory protein (SVS)2, SVS5, SVS4,* and *Deoxyribonuclease II beta (Dnase2b)*. Data are presented as column graphs of log fold-change (logFC) with the dashed line indicating the logFC threshold used to identify differentially regulated genes (logFC ≥0.58 or ≤ − 0.58). **B** RNA-sequencing data was validated using quantitative PCR on *Cfb* (n = 5 individual mice per treatment group). Data was processed using the delta C(t) method using *Peptidyl-prolyl cis-trans isomerase A (Ppia)* as the reference gene and then normalised to the control group. Data are presented as a column graphs with mean ± SEM values. Corresponding transcriptomics data is overlaid onto these data using a line graph. Data were analysed by non-parametric Mann Whitney U test. * *p* ≤ 0.05 indicates statistical significance when comparing acrylamide to control

We have recently demonstrated that a major consequence of exposing mice to acrylamide is a reduction in seminal vesicle secretions accompanied by a change in the tissue expression of a subset of secretory proteins [2]. To provide molecular insight into this phenomenon, the curated seminal vesicle transcriptomic data were assessed for evidence of altered expression profiles among the 70 genes encoding secretory proteins (Additional file 1, Table A2). Notably, only one of these genes (*Complement factor b* (*Cfb*, 2.45-fold increase, *p.adj* = 7.04E-02, Fig. 8A) met the fold-change and significance threshold criteria to be considered as dysregulated; a result that was validated by quantitative PCR amplification of *Cfb* (Fig. 8B, 2.1-fold increase, *p* = 7.9E-03). Aside from *Cfb*, subtle yet significant reductions were recorded in the expression of genes encoding several of the most abundant seminal vesicle secretory proteins, including *Svs2* (21% decrease, *p.adj* = 2.12E-02)*, Svs5* (21% decrease, *p.adj* = 2.10E-02)*, Svs4* (23% decrease, *p.adj* = 6.88E-03) and *Dnase2b* (23% decrease, *p.adj* = 1.77E-02) (Fig. 8A).

## Discussion

Seminal vesicles are the major accessory gland contributing to seminal plasma in most mammalian species. Their secretions promote reproductive success by assisting sperm delivery to the site of fertilisation and modulating the post-copulatory female reproductive tract environment to promote support of embryo development and implantation [4–7]. Despite the physiological significance of the seminal vesicles, a mechanistic understanding of their fundamental biology is lacking. Accordingly, here we provide the first detailed bioinformatic analysis of transcriptomics data from mouse seminal vesicle tissue and additionally, exploit a well-established acute acrylamide exposure model [2, 25–27] to explore how seminal vesicle genes respond to this reproductive toxicant.

The principal function of the seminal vesicles is to manufacture and secrete bioactive factors, leading to the creation of a complex seminal fluid milieu that promotes reproductive success [9, 24, 48]. Seminal vesicle secretions contain a diverse array of proteins, enzymes, mucus, vitamins, amino acids, ion minerals, flavins, and hormones [10]. Notably, the protein profile of seminal vesicle secretions is dominated by a small subset of highly abundant proteins, and a multitude of lower abundance proteins [11, 31, 48]. Consistent with this, the 15,304 genes detected in the mouse seminal vesicles transcriptome were highly biased toward a small subset of dominant genes. Specifically, the three most abundant seminal vesicle genes (*Svs2*, *Svs5* and *Svs4*) had average TPM expression values equivalent to the entire subset of remaining genes. Reflecting the deficit in knowledge of seminal vesicle genes, as many as 3% of all detected genes were incompletely annotated, including three of the 20 most abundant genes that are listed as Riken genes. Notably, our seminal vesicle transcriptomic data held high conservation (80% identity between transcriptomes) with a recently published seminal vesicle transcriptomic analysis undertaken on adult seminal vesicle tissue from mice exposed to diethylstilbestrol or vehicle control [16]. However, Li et al. [16], focused on the cellular pathways influenced by diethylstilbestrol in seminal vesicle tissue and did not undertake bioinformatics of the genes expressed in this tissue under normal physiological conditions [16].

Genes encoding seminal vesicle secretory proteins [23, 31, 32] were among the most abundant detected in seminal vesicle tissue, with 28% of characterised seminal vesicle fluid proteins encoded by genes detected in the seminal vesicle top 50. Unsurprisingly, seven of the ten most highly abundant genes were identified as members of the *Svs* family, whose presence in seminal vesicle fluid is primarily associated with formation of the copulatory plug [24]. Other highly abundant genes included several encoding well-characterised secretory proteins such as *Sva*, *Pate4, Spinkl*, and *Spink1,* which have established roles in modulation of sperm fertilising ability, copulatory plug formation, sperm motility, and/or sperm capacitation [49–54], with markedly higher expression of these genes observed in the seminal vesicles compared to other tissue types. TGM4 was previously reported to be secreted from the prostate and coagulating gland where it interacts with SVS proteins to facilitate copulatory plug formation [24], and here we extend that finding to show that *Tgm4* genes are also highly abundant in the seminal vesicles, a finding consistent with our recent proteomic analysis [2]. Another highly abundant gene of note was *Cd52 antigen* (*Cd52*), which although not previously detected in the seminal vesicle fluid proteome [11, 31, 48], has been shown to influence the female reproductive tract immune environment through sialylation of sperm [55].

Consistent with the view that reproductive hormones are pivotal for seminal vesicle development and function [10, 16, 18, 19, 21], endocrine associated pathways and biological functions were predicted from functional annotation of the seminal vesicle transcriptome. Additionally, RICTOR, a component of the mTORC2 signalling pathway, with well-established roles in protein production and secretion, as well as epithelial cell function [56–64] was predicted as a central upstream regulator of the seminal vesicles. This finding, which aligns with our recent proteomic analyses [2], warrants further consideration of the potentially influential role of RICTOR in seminal vesicle function. Beyond RICTOR, detailed annotation of our combined seminal vesicle transcriptomic and proteomic datasets [2] confirmed a high level of conservation in the predicted functional capacity of the seminal vesicle tissue. In this context, the primary function of the seminal vesicles appears to rest with gene transcription and protein synthesis, presumably allowing this tissue to rapidly initiate cell fate decisions that assist in the maintenance of normal reproductive physiology [65–67].

In recent years, it has been demonstrated that exposure to paternal stressors such as obesity, low-protein diets, and endocrine disrupting compound exposure, have major downstream effects on seminal vesicles and their secretory function [13–16, 68]. These studies build on evidence that the composition of seminal vesicle secretions has important consequences for fertility, fecundity, and offspring phenotype [4, 69]. Indeed, seminal vesicles from diabetic rodents [70] or those subjected to psychological stress in the form of restraint and forced swimming [13], exhibit atrophy and histological abnormalities, characteristic of reduced secretory capacity. Whether a reduction in the volume of seminal vesicle secretions has a physiological consequence remains unknown but, in both rodents and primates, alterations to seminal vesicle secretory capacity has repercussions for a male’s fertility and fecundity [4, 19, 71–73]. To expand molecular understanding of the physiological basis of seminal vesicle responsiveness to environmental factors, we assessed global changes in the seminal vesicle transcriptome elicited by acute challenge with the reproductive toxicant acrylamide [2, 25–27].

Using this model, we have previously demonstrated that the adverse impacts of acrylamide exposure on male reproductive function and fetal development are mediated by effects on sperm quality, whereby acrylamide exposure at any stage of sperm development leads to elevated levels of DNA damage. In contrast, exposure only during epididymal transit causes a substantial increase in fetal loss [25–27]. Our recent data also raise the prospect that paternal stress signals mediated by acrylamide exposure may be due to effects on seminal vesicle physiology and secretory capacity [2]. These combined data suggest that mechanisms independent of sperm DNA damage are responsible for the increased rate of fetal loss observed following acrylamide exposure and position both the epididymis [74–76] and seminal vesicles [2, 4, 15] as key transmitters of paternal exposure load. Here, we demonstrate that acrylamide exposure leads to subtle but potentially important changes in the seminal vesicle transcriptome with the identification of 70 differentially regulated genes resulting from this insult. Functional annotation of the impact of acrylamide on seminal vesicle tissue showed consistency between transcriptomic and proteomic analyses, with biological functions associated with gene/protein synthesis and cellular survival dysregulated following acrylamide exposure [2]. However, a key transcriptomic response that was not obvious in the tissue proteome was the induction of a wide array of immune associated genes, cellular networks, and signalling pathways. This disparity is likely explained by the fact that immune associated proteins, particularly cytokines, are challenging to detect using mass spectrometry due to their low molecular weight and relatively low abundance, necessitating the need for targeted proteomic approaches to detect these molecules [77, 78]. Alternatively, post-transcriptional regulatory mechanisms such as those driven by small RNAs (e.g. miRNA) could contribute to prevent the synthesis of immune associated proteins. Irrespective, immune changes are hallmarks of other cellular models, wherein acrylamide exposure leads to the production of reactive oxygen species and inflammation [79–81]. Indeed, our own studies in the male reproductive tract show that acrylamide exposure leads to the production of reactive oxygen species and the consequent generation of oxidative stress [2, 25, 28, 29, 82], in a manner that would be expected to negatively impact fertility [83]. This is perhaps unsurprising given that the detoxification of acrylamide is initiated via an oxidative catabolic reaction catalysed by the cytochrome P450 (CYP) enzyme CYP2E1, and leading to the formation of a highly reactive glycidamide metabolite [84].

Inflammation plays an important role in cells and tissues to promote phenotypic fluidity in response to injury [85], where the induction of oxidative stress, production of inflammatory mediators, and influx of leukocytes play critical roles in returning the tissue to normal homeostasis [86, 87]. It is therefore noteworthy that several of the differentially regulated genes identified in the IPA analyses, in addition to the predicted upstream regulators (*Csf2*, *Ifng*, and *Fas*), play critical roles in regulation of wound healing and tissue repair [88–90]. For example, IPA identified the upstream regulator *Egr1,* from the EGR family of zinc finger transcription factors, as a potent regulator of the immune response and important modulator of cell growth, differentiation, and survival [91]. Deficiency of members of the *Egr* gene family in the mouse leads to seminal vesicle atrophy [92]. Additionally, genes dysregulated following acrylamide exposure and linked to tissue repair and remodelling included *Ccl8* [93], *C4b* [94], *Col5a1* [95], and *Ido* [96]. Moreover, our IPA analyses identified 25 of dysregulated genes associated with inflammation functions, signalling pathways or disease states.

While there is limited information about the leukocyte profile of seminal vesicle tissue, studies in humans have shown that macrophages and T cells are abundant within this tissue [97, 98]. Similarly, the major leukocyte population in mouse seminal vesicles are macrophages [99]. Notably, dysregulation of signalling pathways involving both macrophages (agranulocyte adhesion and diapedesis) and T cells (T-helper cell differentiation and altered T and B cell signalling) were identified by IPA as prominent cellular responses impacted by acrylamide exposure. Additionally, our data demonstrates that acrylamide activates *Csf2,* a putative upstream regulator that exerts profound effects on the function of several types of leukocytes, including macrophages [100]. While little is known about the roles of T cells in the seminal vesicles, depletion of macrophages has been linked to reductions in seminal vesicle weight and secretory activity [101]; a response that is, at least in part, attributable to reduced circulating steroids [101, 102]. However, given that acrylamide exposure also reduces seminal vesicle secretory capacity [2], these data raise the prospect the macrophages may fulfil a previously unappreciated role in seminal vesicle secretory function. Such findings draw interesting parallels with the function of macrophages in regulating epithelial cell structure, integrity, and secretory activity in other mucosal surfaces, including the mammary gland and uterus [103–105]. It will therefore be of value for future studies to explore the influence of resident immune cells, particularly macrophages, on seminal vesicle biology.

While the functional consequences of the altered immune environment in the seminal vesicles following acrylamide exposure remain to be deciphered, our previous studies have shown that an equivalent treatment regimen causes alterations in the secretory capacity and composition of seminal vesicle secreted proteins [2]. A link between inflammation and the composition of seminal vesicle fluid is biologically relevant as several studies have demonstrated that infection and inflammation cause altered seminal plasma cytokine content in humans [106–109]. An example is IFNG, which is present in seminal vesicle fluid of mice [110] and detected in seminal plasma of humans [109]. IFNG expression is increased in response to reproductive tract infection or microbial dysbiosis [106, 111], and thereafter adversely impacts both sperm function and female immune adaptation for pregnancy [108]. Here, our IPA analysis of the differentially expressed genes identified the complement system signalling as a significant pathway in response to acrylamide. Within this signalling pathway, we observed increased expression of *Cfb*, a complement molecule detected in the seminal fluid of bull [112] and mice [32], which is proposed to mediate interactions between sperm and female reproductive tract neutrophils [113, 114]. Additionally, *DNase2b*, a highly abundant DNase enzyme reduced following acrylamide exposure is also implicated in regulation of sperm neutrophil interactions [115], while reductions in *Svs2, 4–5* may exert influence over copulatory plug formation [24] and sperm survival in the female reproductive tract [71]. While these changes are subtle, they conceivably could influence male reproductive fitness in a manner that has consequences for male competition for reproductive success, as has been postulated in studies on ejaculate composition in the house mouse [6, 11, 23]. Indeed, altered seminal vesicle composition might well contribute to the reduced fecundity and elevated fetal loss that occurs in pregnancies sired by mice after acute acrylamide exposure, that to date has been attributed to the direct impact of acrylamide on sperm-borne stress signals [25–27]. However, whether changes in the abundance of seminal vesicle genes correlate with the secretory profile of their gene product, and the impact of this on the various biological functions of seminal plasma, remain to be determined. Future studies are therefore needed to elucidate the specific impact of acrylamide exposure on seminal vesicle secreted proteins that protect sperm integrity after ejaculation, and cytokines and signalling molecules that interact with the female reproductive tract immune response after mating.

## Conclusions

In summary, here we report the first comprehensive characterisation of mouse seminal vesicle genes under normal physiological conditions as well as defining the response of this tissue to the potent reproductive toxicant, acrylamide. These data add to an emerging body of literature demonstrating that the seminal vesicles, similar to other tissues comprising the male reproductive tract and accessory organs, are sensitive to environmental factors and exposures [4, 6, 7, 15, 68], and respond in a manner that may have consequences for fertility, fetal development and later offspring health. Advancing our mechanistic understanding of seminal vesicle physiology will be crucial for understanding the factors that impinge on seminal fluid composition, and the implications of its perturbation for reproductive success and programming of offspring health.

## Methods

### Ethics approval

All experimental procedures involving mice were conducted with the approval of the University of Newcastle Animal Care and Ethics Committee (approval number A-2017-726) and accorded with the Australian Code of Practice for the Care and Use of Experimental Animals.

### Mice

Male outbred Swiss mice were obtained from a breeding colony held at the University of Newcastle central animal facility and maintained according to the recommendations prescribed by the Animal Care and Ethics Committee. Mice were housed under a controlled lighting regimen (12 h light: 12 h dark) at 21 °C – 22 °C and supplied with food and water ad libitum.

### Chemicals and reagents

All reagents were purchased from Merck (Darmstadt, Germany), unless otherwise specified.

### Acrylamide treatment regimen and tissue collection

Mice (8–12 weeks old) received an intra-peritoneal injection of acrylamide (25 mg/kg body weight) or vehicle alone (phosphate buffered saline (PBS) 137 mM NaCl, 2.7 mM KCl, 8 mM Na_2_HPO_4_, and 2 mM KH_2_PO_4,_ pH 7.4) each morning for five consecutive days following our established method [2, 25]. Mice were euthanised 72 h following the final injection and seminal vesicles were dissected following careful removal of prostate tissue. Seminal vesicle tissue was weighed and washed thoroughly in Tris-buffered saline (TBS, 50 mM Tris-Cl, 150 mM NaCl, in mass spectrometry (MS) grade H_2_O, pH 7.6). Seminal vesicle fluid was removed by gently squeezing seminal vesicle tissue using curved forceps. Tissue was snap frozen in liquid nitrogen and stored at − 80 °C for transcriptomic analysis.

### RNA extraction

Seminal vesicle samples were homogenised using the FastPrep-24 5G homogeniser (MP Biomedicals, Irvine, CA) with the Cool Prep Adaptor (2 × 1 min, 4.0 m/s, at 4 °C) in Trizol reagent (ThermoFisher Scientific, Waltham, MA). Total RNA was extracted using the Trizol RNA method and DNase treated using RQ1 RNase free DNase (Promega, Madison, WI), following the manufacturer’s instructions. RNA was quantified using a NanoDrop Lite Spectrophotometer (ThermoFisher Scientific) and RNA integrity was assessed using an Agilent Bioanalyzer (Agilent Technologies, Santa Clara, CA). RNA with a RIN > 7 was used in this study. Genomic DNA removal was assessed as described [107]. RNA was stored at − 80 °C prior to use.

### Transcriptomics analysis

RNA-sequencing analysis was performed at BGI Genomics (Shenzhen, China) using DNBseq technology. Poly-A libraries were constructed by initially purifying poly-A mRNAs using poly-T oligo attached magnetic beads. Poly-A mRNAs were fragmented using divalent cations and the cleaved RNA was processed through first strand cDNA synthesis using reverse transcriptase and random primers, followed by second strand cDNA synthesis using polymerase I and RNase H. Second strand cDNA was then purified, and DNA nanoballs were generated with the libraries by rolling circle replication and samples were loaded into patterned nanoarrays to generate 100 bp paired end reads through the DNBseq platform. For each library, a minimum of 67 million paired end raw reads were produced (Additional file 2, Fig. A1*C*). All of the generated raw sequencing reads were filtered using SOAPnuke [116] to remove reads with adaptors, reads with more than 0.1% unknown bases, and low-quality reads (more than 40% base quality < 20). Trimmed reads (Additional file 2, Fig. A1*D*)) accounted for > 93.5% total raw reads and were stored in FASTQ format and were initially mapped onto the mouse reference genome (mm10) using Hisat2. Novel genes were predicted using Stringtie [117] and Cuffcompare (Cufflinks tools) [118], and CPC [119] was used to predict the coding potential of novel genes before obtaining a complete reference to be used in downstream analyses by merging coding novel genes with reference genes. Autosomal gene expression levels were calculated using RSEM [120]. To assess the impact of acrylamide exposure on the seminal vesicle transcriptome, differential regulation of genes was assessed between acrylamide and control groups using DEseq2 [121]. Scatter plots of log transformed normalised abundances (Additional file 2, Fig. A1*A-B*) were then generated, and Pearson correlation (r2) were calculated, while global analysis of the data was undertaken to generate a Principal component analysis plot. For a global illustration of protein abundance compared to fold-change, an MA plot was generated using Microsoft Excel.

### Phenotype and seminal vesicle fluid comparisons

The seminal vesicle fluid transcriptome was searched against an exported gene list consisting of the full complement of Mouse Genome Informatics [122] and the International Mouse Phenotyping Consortium [123] datasets to identify those genes expressed in the seminal vesicle that are also associated with either male fertility or seminal vesicle phenotypes. To further complement these analyses, proteomic datasets from three seminal vesicle fluid proteomic characterisation papers [23, 31, 32] were obtained and filtered for proteins identified in 1/3 studies. Proteins that were identified in at least one manuscript were then used to form a list of proteins expressed in mouse seminal vesicle fluid. Seminal vesicle fluid proteins were then mapped to their corresponding mouse genes resulting in a final list of 81 genes. This list was then compared to our seminal vesicle transcriptome. Seminal vesicle gene expression (TPM) was used to determine the ranking order of abundance of genes whose products are secreted into seminal vesicle fluid.

### Comparative transcriptomics analysis

To compare the seminal vesicle transcriptome to previously published mouse transcriptomic analyses, we obtained data from a recent large-scale discovery study of male reproductive tract specific genes [33], which presented data in TPM. These transcriptomic data included testis, spermatogonia, Sertoli cell, caput epididymis, corpus epididymis, cauda epididymis, as well as the transcriptomes of non-reproductive tissues, including adipose tissue, adrenal gland, bone marrow, cerebral cortex, colon, duodenum, heart, kidney, liver, lung, pancreas, skeletal muscle, small intestine and stomach [33, 124–127] and were log2 TPM data collated from Additional file 4, Table S4 in [33]. From our seminal vesicle transcriptome, RSEM calculated autosomal gene expression levels [120] were normalised using the TPM approach [120] and for the comparison to other cell types was similarly converted to log2 values. Due to their abundance in the seminal vesicle transcriptome, we then selected those highly abundant genes whose products are also detected in seminal vesicle fluid proteomic analyses [23, 31, 32] and performed a comparative analysis to determine the expression of seminal vesicle genes across tissue types.

### Ingenuity pathway analysis

Ingenuity Pathway Analysis (IPA) software (Qiagen, Hilden, Germany) was used to analyse the refined seminal vesicle transcriptomic data as previously described [36]. Canonical pathways, upstream regulators, gene networks and disease and function analyses generated using IPA were assessed using; *p*-value that is an enrichment measurement of the overlapping proteins from the dataset in a particular pathway, function or regulator, and; *Z*-score that is a prediction scoring system of activation or inhibition based upon statistically significant patterns in the dataset and prior biological knowledge manually curated in the Ingenuity Knowledge Base [128]. To elucidate the most significant changes in our analyses, we applied a stringency criteria *p*-value ≤0.05 and a *Z*-score of (inhibition) -2 ≤ Z ≥ 2 (activated). Networks of interest were constructed in IPA, organised by subcellular location and redrawn using BioRender software (BioRender, San Francisco, USA).

### Reverse transcription and PCR

Total cellular RNA (1 μg) was reverse transcribed to generate cDNA from 500 ng Oligo (dT) primed RNA in a solution containing 1 × Moloney Murine Leukaemia Virus (M-MLV) reaction buffer, 10 mM dNTPs, 25 U recombinant RNasin Ribonuclease Inhibitor, and 200 U M-MLV Reverse Transcriptase (all Promega Corporation, Madison, WI). For both conventional PCR and quantitative PCR (qPCR), primer pairs specific (Additional file 1, Table A9) for gene sequences were optimised for PCR in validation experiments using control mouse seminal vesicle cDNA. PCR products were visualised on 1% w/v agarose gels using a Bio-Rad Chemidoc MP Imaging System (Bio-Rad Laboratories, Hercules, CA) and sequence specificity was confirmed using Sanger sequencing (Australian Genome Research Facility, Sydney, Australia) of PCR products purified using the Wizard SV Gel and PCR Clean-Up System (Promega Corporation) as per manufacturer’s instructions.

Conventional PCR was performed on control seminal vesicle cDNA (*n* = 4 biological replicates / treatment groups) in duplicate using GoTaq DNA Polymerase (Promega) according to the manufacturer’s instructions on an Eppendorf Mastercycler Pro Thermal Cycler (Merck). Reactions were performed on cDNA equivalent to 100 ng containing PCR primers (Supplemental Table S1). PCR amplification was conducted using the following conditions: 95 °C for 5 min followed by 40 cycles of 94 °C for 45 s, 55 °C to 68 °C (Additional file 1, Table A9) for 45 s and 72 °C for 1 min, with a final extension step of 72 °C for 10 min. PCR products were visualised on 1% agarose gels using a Bio-Rad Chemidoc MP Imaging System (Bio-Rad Laboratories).

qPCR was performed in duplicate from n = 4–6 samples / treatment group using SYBR Green GoTaq qPCR master mix (Promega) according to manufacturer’s instructions on a Roche Light Cycler 96 (Roche Holding AG, Basel Switzerland). Reactions were performed on cDNA equivalent to 10 ng of total cDNA containing PCR primers (Additional file 1, Table A9). PCR amplification used the following conditions: 95 °C for 2 min followed by 40 cycles of 95 °C for 15 s and 58–66 °C (Additional file 1, Table A9) for 1 min. The delta C(t) method [129] was then used to calculate messenger RNA abundance normalised to *Peptidyl-prolyl cis-trans isomerase A* (*Ppia*), which was identified from a subset of reference genes as the most suitable in this experiment using the Bestkeeper reference gene selection algorithm [130].

### Statistical analysis

All analyses in this study were performed using biological replicates of seminal vesicle tissue where a biological replicate is defined as seminal vesicle tissue collected from an individual mouse. Transcriptomic analyses were performed using seminal vesicle tissue collected from n = 4 biological replicates / treatment group. These data were assessed by DESeq2 [121] and genes were defined as differentially expressed when they met the criteria of a fold-change ≥1.5 or ≤ 0.67, and a false discovery rate adjusted *p* value (*p.adj*) ≤ 0.1. For qPCR (n = 4–6 biological replicates / treatment group) confirmation of transcriptomic data, data were initially assessed for normality using the Shapiro-Wilk normality test. Data normally distributed was assessed by unpaired t-test while, data not normally distributed was assessed by a non-parametric Mann-Whitney U test, to detect differences between treatment groups where *p* < 0.05 deemed statistically different. Graphical data, including proportional bar charts, heatmaps, volcano plots and pie charts were prepared using GraphPad Prism version 9.0.0 for Windows (GraphPad Software, San Diego, CA).

## Supplementary Information

**Additional file 1: Tables A1-A9.** This additional file contains all supplemental tables for this manuscript.

**Additional file 2: Figure A1.** This additional file contains supplemental figure A1 for this manuscript.

**Additional file 3: Figure A2.** This additional file contains supplemental figure A2 for this manuscript.

## Data Availability

The data supporting the conclusions of this article are available in the NCBI sequence read archive repository at GSE183632, https://www.ncbi.nlm.nih.gov/geo/query/acc.cgi?acc=GSE183632. Other transcriptomics data used in this study were obtained from [16] with permission from the authors, and accessed from Additional file 4, Table S4 of [33].
