# Supplementary figures and images for: Transcriptomic analysis of the seminal vesicle response to the reproductive toxicant acrylamide
Source: BMC Genomics. 2021 Oct 8;22:728. doi: 10.1186/s12864-021-07951-1 (PMC8499523; doi:10.1186/s12864-021-07951-1)

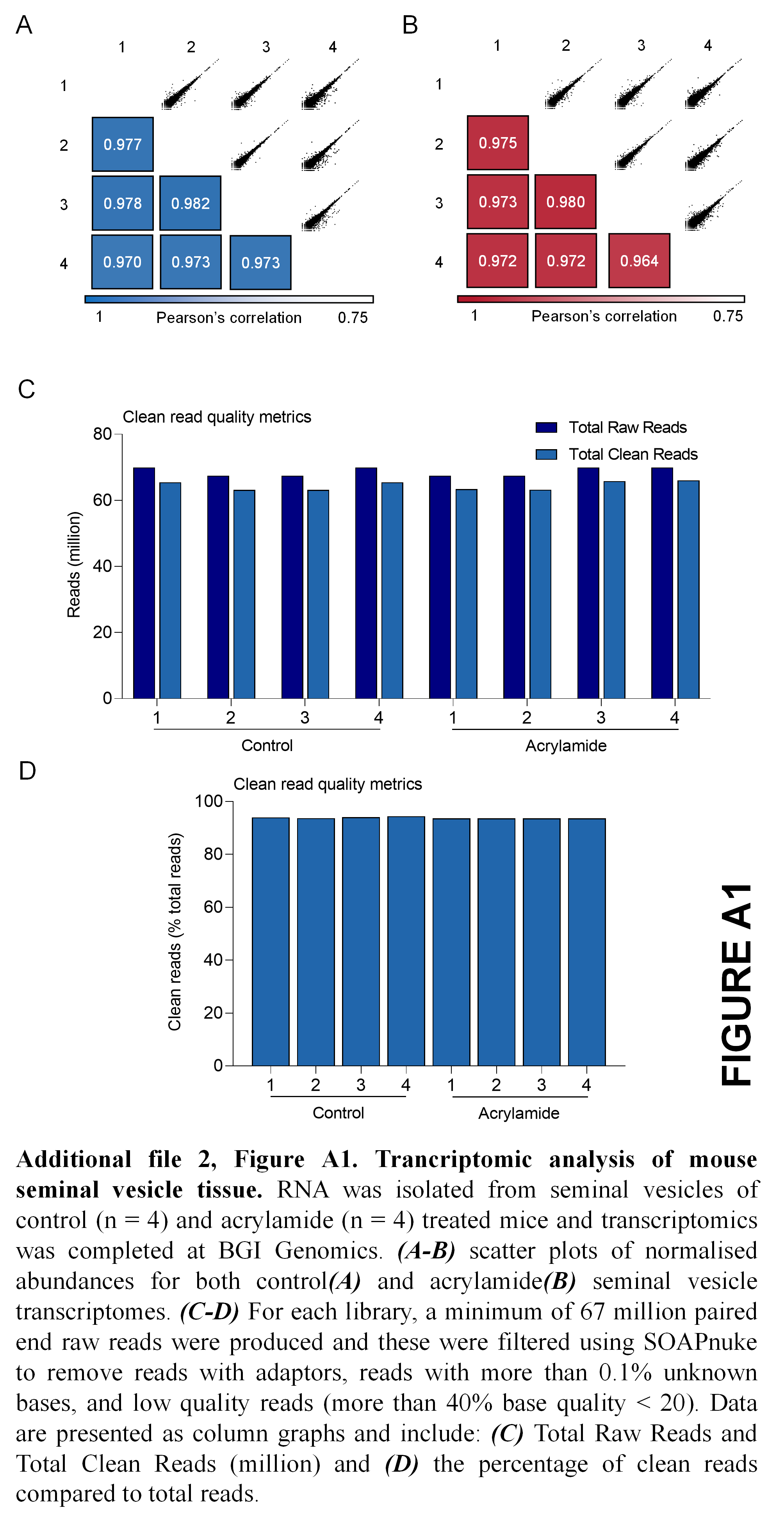

Supplement: Supplementary file 2 — Additional file 2: Figure A1. This additional file contains supplemental figure A1 for this manuscript. [file 12864_2021_7951_MOESM2_ESM.tif]

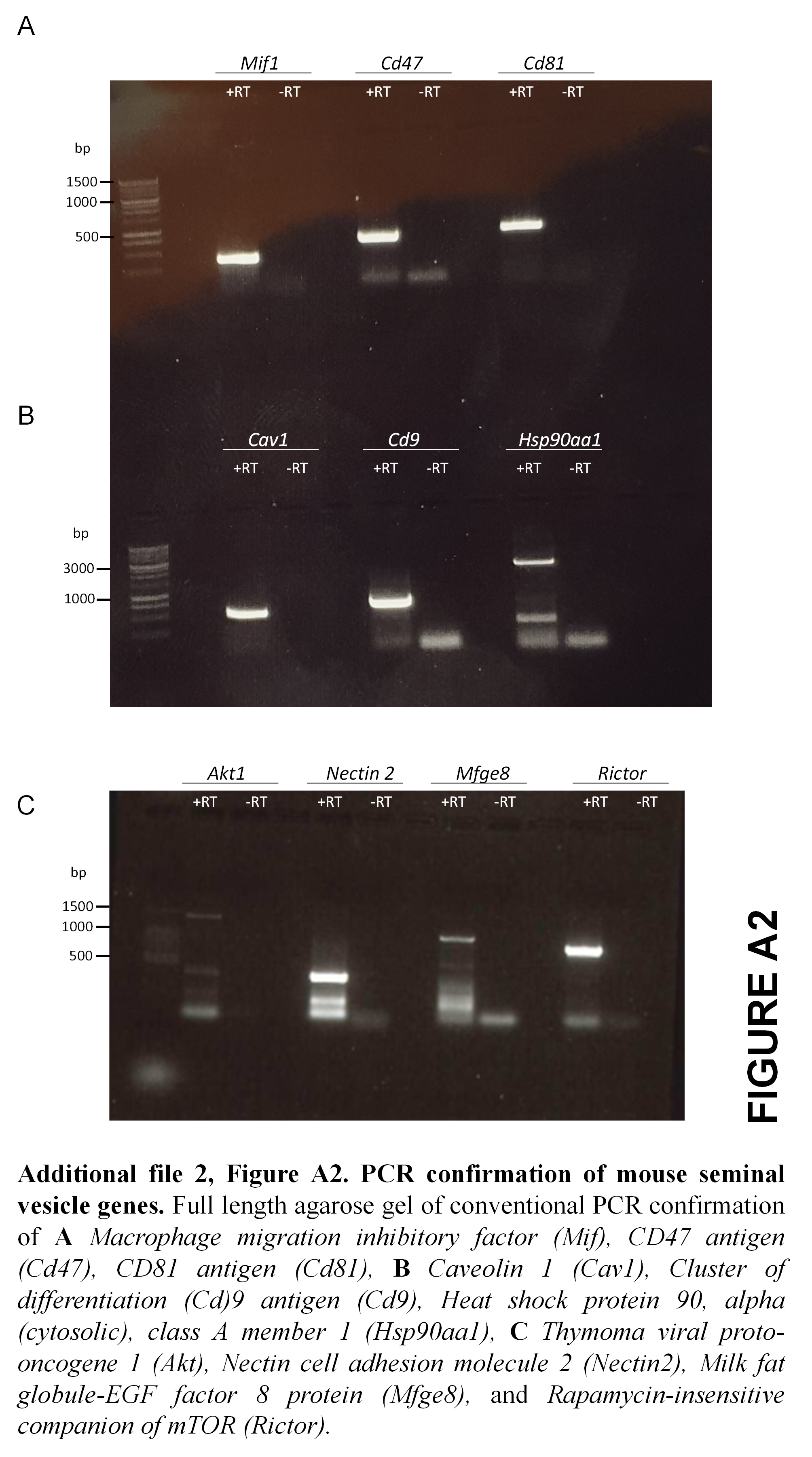

Supplement: Supplementary file 3 — Additional file 3: Figure A2. This additional file contains supplemental figure A2 for this manuscript. [file 12864_2021_7951_MOESM3_ESM.tif]
